# Trombo flutuante em veia femoral

**DOI:** 10.1590/1677-5449.005817

**Published:** 2017

**Authors:** Matheus Bertanha, Rafael Elias Farres Pimenta, Gustavo Muçouçah Sampaio Brandão, Marcone Lima Sobreira, Regina Moura, Rodrigo Gibin Jaldin, Paula Angeleli Bueno de Camargo, Winston Bonetti Yoshida

**Affiliations:** 1 Universidade Estadual Paulista Júlio de Mesquita Filho – UNESP, Faculdade de Medicina de Botucatu, Departamento de Cirurgia e Ortopedia, Botucatu, SP, Brasil.

**Keywords:** trombose venosa, trombectomia, anticoagulantes, fibrinólise, embolia pulmonar

## Abstract

O trombo venoso flutuante em veia femoral é um tipo de trombo com alto potencial de embolização pulmonar. Entretanto, ainda é controversa a conduta mais apropriada nesses casos. Tratamentos clínicos com anticoagulantes ou fibrinolíticos e trombectomias abertas ou por meio de dispositivos endovasculares vêm sendo empregados ainda sem um critério de indicação bem definido. Apresentamos três casos clínicos de trombos flutuantes em veia femoral, de etiologias distintas, cujos tratamentos e respectivas evoluções serão discutidos.

## INTRODUÇÃO

O trombo venoso flutuante em veia femoral é um tipo de trombo com alto potencial de embolização pulmonar^1^^-^ 4. Ele é caracterizado por uma massa trombótica não aderida à parede venosa, que avança para dentro da luz de uma veia profunda coletora^5^. Segundo Voet et al.^6^, a prevalência de trombo flutuante encontrada em estudo de 44 casos de trombose venosa profunda (TVP) proximal foi de 18%, sendo a localização mais comum na veia femoral (38% na junção safenofemoral), seguida da veia poplítea (26% na junção da veia safena parva) e da veia ilíaca externa (15%). Nessa casuística, 87% dos trombos desapareceram após 3 meses de tratamento anticoagulante no seguimento com ultrassom dúplex (USD), independentemente de sua localização. O trombo flutuante pode ser encontrado também em pacientes que apresentam apenas trombose venosa superficial (TVS) isolada^7^, em que pode se estender para uma veia profunda^4^, mas também vem sendo referido como uma complicação do tratamento de veias safenas insuficientes com *laser* endovenoso^8^. No estudo de Chengelis et al.^9^, entre 263 casos de TVS, 30 pacientes tinham também TVP, dos quais 12 pacientes apresentavam trombo flutuante na veia femoral. Sobreira et al.^10^ encontraram 21,7% de TVP e 28,3% de embolia pulmonar (EP) documentada em 60 casos de TVS.

Não há um consenso bem estabelecido quanto ao melhor tratamento para esses casos de forma que possa prevenir a EP e a recorrência de TVP. Neste desafio terapêutico, apresentamos três casos de trombo flutuante em veia femoral, os tratamentos realizados e as respectivas evoluções.

## PARTE I – SITUAÇÕES CLÍNICAS

**Figura 1:**
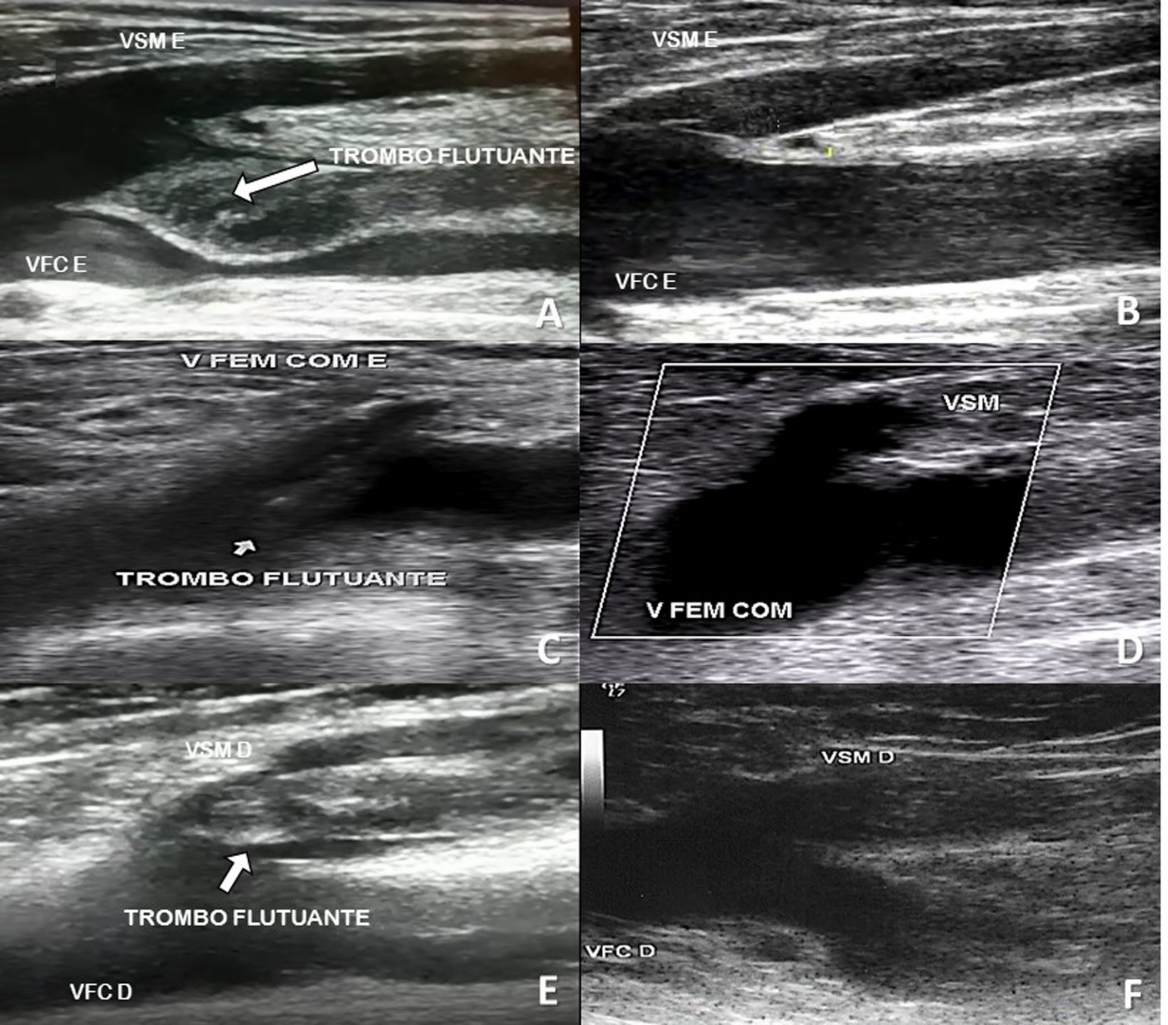
Ultrassom dúplex venoso dos três casos apresentados. **A)** Caso 1 - Trombo flutuante em veia femoral comum esquerda; **B)** Caso 1 - Recanalização da veia femoral esquerda; **C)** Caso 2 - Trombo flutuante desde a junção safenofemoral até a veia femoral esquerda; **D)** Caso 2 - Recanalização completa da junção safenofemoral esquerda; **E)** Caso 3 - Trombo flutuante na junção safenofemoral direita; **F)** Caso 3 - Recanalização completa da junção safenofemoral direita. D = direito; E = esquerdo; VFC = veia femoral comum; VSM = veia safena magna.

**Caso 1 -** Paciente com 69 anos, do sexo masculino, apresentou quadro súbito de dispneia com dor torácica, com sinais vitais dentro da normalidade. A suspeita clínica de EP foi confirmada por angiotomografia. Tinha antecedentes de hipertensão arterial, retocolite ulcerativa e episódio prévio de TVP em membro inferior esquerdo vários anos antes e que não soube precisar, sendo que naquela época recebeu anticoagulação com varfarina por 1 ano, suspensa após esse período. Os pulsos periféricos estavam palpáveis. O USD venoso realizado para investigação de fonte emboligênica mostrou trombo flutuante em veia femoral, além de TVP em toda veia femoral e poplítea esquerda (Figura 1A).

**Caso 2 -** Paciente com 50 anos, do sexo masculino, apresentou queixa de dor e edema do membro inferior esquerdo com uma semana de evolução, que iniciou após realização de esforço físico mais intenso (relacionado ao trabalho). Não apresentava qualquer outra comorbidade associada. Negou tabagismo, alcoolismo, cirurgias prévias, imobilizações, traumas, obesidade e restrições à deambulação. Não apresentava antecedentes familiares de EP, TVP ou TVS. Ao exame físico, apresentava-se normotenso, com edema em membro inferior esquerdo (+++/4), e apresentava ainda hiperemia, calor e dor no trajeto da veia safena magna, desde a porção proximal da perna até a porção proximal da coxa, em membro inferior esquerdo, sem estigmas de insuficiência venosa crônica grave, exceto por varizes esparsas não comunicantes. Os pulsos periféricos estavam palpáveis e havia empastamento muscular na região da panturrilha e coxa esquerda. Foi realizado USD venoso no membro inferior esquerdo, que evidenciou veia safena magna incompressível, sem fluxo sanguíneo e com conteúdo ecogênico (trombo) em seu interior. O trombo se estendia desde a porção média da perna até a junção safenofemoral, onde “mergulhava” para o interior do lúmen da veia femoral comum e apresentava-se “flutuante” (Figura 1C; Vídeo 1 do Material Suplementar), com movimento síncrono com a pulsação arterial. As demais veias superficiais e profundas estavam normais. A conclusão diagnóstica do exame foi tromboflebite ascendente da veia safena magna e trombo flutuante da veia femoral comum, em membro inferior esquerdo.

**Caso 3 -** Paciente com 58 anos, do sexo feminino, com varizes e insuficiência venosa crônica de longa data e com úlcera venosa no membro inferior direito. A úlcera se originou de um trauma na região maleolar medial desse membro (arranhão), com consequente infecção local, que foi tratada com antibióticos (cefalexina). Teve reação alérgica aos antibióticos e ficou em repouso tratando a ferida com curativos locais. Tinha dor intensa no local, que piorava quando ficava em pé. Após 15 dias em tratamento conservador, procurou atendimento médico especializado, agora apresentando também hiperemia, dor e calor no trajeto da veia safena magna direita. Ao exame clínico estava normotensa, com pulsos arteriais distais presentes, úlcera venosa ativa com tecido de granulação, de 4 cm de diâmetro, na face medial da perna direita (CEAP 6). O USD venoso mostrou TVS da veia safena magna direita, que se estendia por todo seu trajeto até a junção safenofemoral, sendo observado trombo flutuante no limite da junção safenofemoral, móvel com batimentos cardíacos (Figura 1E).

As possíveis opções de tratamento consideradas para esses casos incluem: tratamento anticoagulante^3^^,^ 11; tratamento fibrinolítico ou trombólise^12^; ligadura venosa ou plicatura venosa^1^; trombectomia venosa^11^; trombectomia aspirativa percutânea^11^; e filtro de veia cava inferior (FVC)^11^.

## PARTE II – O QUE FOI FEITO

**Caso 1 -** Foi tratado com anticoagulação plena durante a internação hospitalar, com associação de heparina não fracionada e varfarina até obtenção de nível terapêutico da razão normalizada internacional (RNI entre 2-3). Devido à ocorrência de EP e trombo flutuante, optou-se por implante de um FVC removível. O USD foi realizado no quarto dia de internação hospitalar e evidenciou ausência de trombos no FVC, que se apresentava bem locado, e manutenção do quadro ultrassonográfico prévio. Recebeu alta hospitalar após 5 dias com prescrição de varfarina 5 mg e orientação de seguimento ambulatorial de rotina para conferência dos níveis de anticoagulação. No seguimento de 6 meses, apresentou-se em bom estado clínico e, ao exame de controle com USD venoso, apresentou remissão total do trombo flutuante com sinais de recanalização das veias acometidas por TVP (Figura 1B). O FVC apresentava-se bem locado e sem trombos em seu interior, e não foi removido.

**Caso 2 -** Foi tratado por meio de anticoagulação com enoxaparina sódica associada a varfarina. No quinto dia de tratamento, o paciente retornou para reavaliação e apresentou melhora importante do edema (+/4) e dos sinais flogísticos. Foi suspensa a heparina de baixo peso molecular e mantida a dose de 5 mg de varfarina, com níveis terapêuticos de RNI. O paciente recebeu prescrição de meia elástica e foi orientado a retornar às suas atividades habituais. Na semana seguinte, voltou para nova reavaliação e encontrava-se assintomático, sem edema residual, com discreta hipercromia no trajeto da veia safena magna no 1/3 proximal da perna e distal da coxa. Foi solicitada pesquisa laboratorial para trombofilias. Não houve qualquer intercorrência durante a fase inicial da anticoagulação e até o presente momento (fase de manutenção), e os exames para trombofilias secundárias foram negativos (pesquisa de câncer oculto e síndrome de anticorpos antifosfolípides). O exame de USD venoso realizado após 3 meses do evento clínico mostrou completa recanalização da veia femoral comum e da veia safena magna com remissão total do trombo flutuante (Figura 1D).

**Caso 3 -** Neste caso, a paciente foi tratada com associação de enoxaparina 1 mg/kg de 12/12h (60 mg) e varfarina durante a internação hospitalar. Os exames para trombofilias secundárias foram negativos. Durante o seguimento ambulatorial, manteve-se com nível terapêutico de anticoagulação por 3 meses, sendo então suspensa a anticoagulação. O exame de USD venoso realizado para seguimento após o término da anticoagulação evidenciou completo desaparecimento do trombo flutuante, recanalização da veia safena magna e ausência de sinais de TVP (Figura 1F).

## DISCUSSÃO

Tendo em vista o potencial de autólise dos trombos flutuantes^6^, o tratamento anticoagulante parece ser uma alternativa não invasiva e atrativa, principalmente após a TVS que atinge a junção safenofemoral^13^^,^ 14. Além do efeito antitrombótico óbvio, os anticoagulantes, especialmente as heparinas, possuem atividades anti-inflamatórias que potencializam os seus benefícios^15^. Nos casos de TVS isolada, o consenso do American College of Chest Physicians (ACCP) de 2012 recomendou uso de fondaparinux em dose profilática (2,5 mg/dia) ou heparina de baixo peso molecular por 45 dias^16^. No entanto, na presença de TVP associada, o tratamento deve ser focado nessa doença, com aumento do tempo de anticoagulação. O tratamento inicial deve ser realizado em ambiente hospitalar. O tratamento domiciliar deve ser descartado devido aos altos riscos de complicações relacionadas à possibilidade de não adesão ao tratamento proposto^17^.

As vantagens do tratamento cirúrgico são: alívio sintomático mais rápido e menor tempo de internação hospitalar, o que reduz os custos^13^. No entanto, a simples ligadura do tronco safeno não impede a passagem de trombos pelas perfurantes e tributárias nem diminui o estado de hipercoagulabilidade eventualmente presente^13^. Na abordagem cirúrgica, além da ligadura, recomenda-se venotomia na croça da veia safena e retirada da cabeça do trombo (trombectomia), que se insinua na luz da veia femoral^1^. As complicações desse tipo de procedimento são a ocorrência de hematomas e hemorragias, e o risco de embolia do trombo e recorrência de trombose local^13^. Em geral, preconiza-se tratamento anticoagulante no período pós-operatório^13^.

O tratamento trombolítico dissolve o trombo efetivamente e reduz a síndrome pós-trombótica (RR: 0,66; IC95%: 0,47-0,94), mas está associado a uma frequência maior de complicações hemorrágicas^12^. No estudo ATTRACT^18^^,^ 19, foram randomizados 692 pacientes com TVP proximal para tratamento fibrinolítico ou tratamento convencional (anticoagulantes e meia elástica). Após 2 anos, observou-se que o tratamento fibrinolítico não preveniu a síndrome pós-trombótica, mas reduziu sua gravidade em 25% dos casos (18% *versus* 24%)^18^.

O FVC é indicado em casos de contraindicação ou de complicações dos anticoagulantes, segundo o consenso do ACCP^11^^,^ 16. Não há diretriz para sua indicação nos casos de trombos flutuantes. Entretanto, nos casos de trombos flutuantes extensos envolvendo veias ilíacas e mesmo a veia cava, o uso de FVC pode ser avaliado, devido ao alto risco de EP, em especial na contraindicação ou falha do uso de anticoagulantes.

## CONCLUSÃO

Nos casos de trombose venosa flutuante com trombo que se insinua para a veia femoral, não há uma diretriz específica de tratamento. O tratamento anticoagulante parece ser uma alternativa menos invasiva e eficiente, e cirurgias podem estar associadas a complicações. Em casos com trombos flutuantes mais extensos, envolvendo veias ilíacas ou mesmo a veia cava, embora não haja consenso, o uso de FVC pode ser avaliado, apesar do alto risco de EP, em especial na contraindicação do uso de anticoagulantes.

## Material Suplementar

Este artigo possui material suplementar.

Vídeo 1 Ultrassom dúplex venoso do caso 2 com trombo flutuante na junção safenofemoral direita. O material está disponível como parte do artigo no site: http://www.scielo.br/jvb.
