# Supplementary material for: Trombo flutuante em veia femoral
Source: J Vasc Bras. 2017 Oct-Dec;16(4):314–9. [Article in Portuguese] doi: 10.1590/1677-5449.005817 (PMC5944309; doi:10.1590/1677-5449.005817)
Supplement: Translation

# Translation: Floating thrombi in femoral veins

**Authors:** Matheus Bertanha, Rafael Elias Farres Pimenta, Gustavo Muçouçah Sampaio Brandão, Marcone Lima Sobreira, Regina Moura, Rodrigo Gibin Jaldin, Paula Angeleli Bueno de Camargo, Winston Bonetti Yoshida

**Affiliations:** 1 Universidade Estadual Paulista Júlio de Mesquita Filho – UNESP, Faculdade de Medicina de Botucatu, Departamento de Cirurgia e Ortopedia, Botucatu, SP, Brazil.

**Keywords:** venous thrombosis, thrombectomy, anticoagulants, fibrinolysis, pulmonary embolism

## Abstract

A floating venous thrombus in the femoral vein is a type of thrombus with a high potential for pulmonary embolization. However, the most appropriate management for these cases is still controversial. Clinical treatments, using anticoagulants or fibrinolytics, open thrombectomies, or thrombectomies by means of endovascular devices have all been used, although the criteria for indication of each are not yet defined. We present 3 clinical cases of floating thrombi in femoral veins with different etiologies and discuss their respective treatments and outcomes.

## INTRODUCTION

A floating venous thrombus in the femoral vein is a type of thrombus with a high potential for pulmonary embolization.^1^^-^ 4 It is characterized by a thrombotic mass that is not attached to the venous wall and advances through the lumen of a deep collector vein.^5^ According to Voet et al.,^6^ the prevalence of floating thrombus in a study of 44 cases of proximal deep venous thrombosis (DVT) was 18%, and the most common site was the femoral vein (38% at the saphenofemoral junction), followed by the popliteal vein (26% at the junction with the small saphenous vein) and the external iliac vein (15%). In that patient sample, 87% of thrombi disappeared after 3 months of anticoagulant treatment according to follow-up duplex ultrasound (DUS), irrespective of site. Floating thrombi can also be seen in patients who exhibit only superficial venous thrombosis (SVT),^7^ in whom this can extend to a deep vein,^4^ but it has also been reported as a complication of endovenous laser treatment for incompetent saphenous veins.^8^ Chengelis et al.^9^ conducted a study in which 30 patients out of a total of 263 cases of SVT also had DVT and 12 of these patients had a floating thrombus in the femoral vein. Sobreira et al.^10^ observed a DVT rate of 21.7% and a pulmonary embolism (PE) rate of 28.3% in a series of 60 cases of SVT.

There is no well-established consensus on the best treatment for these cases in order to prevent PE and recurrence of DVT. In this Therapeutic Challenge we present three cases of floating thrombi in femoral veins, their treatments and their outcomes.

## PART I – CLINICAL SITUATIONS

**Case 1 -** A 69-year-old male patient presented with sudden onset dyspnea with chest pain and with vital signs within normal limits. The clinical suspicion of PE was confirmed with angiotomography. He had a history of arterial hypertension, ulcerative rectocolitis, and a previous DVT episode involving the left lower limb several years previously (he was unable to provide an exact date), on which occasion he had been put on anticoagulation with warfarin for 1 year, after which it had been withdrawn. Peripheral pulses were palpable. A venous DUS conducted to investigate the emboligenic source revealed a floating thrombus in the femoral vein, in addition to DVT involving the entire left femoral and popliteal veins (Figure 1A).

**Figure 1:** Venous duplex ultrasound from the three cases described. **A)** Case 1 - Floating thrombus in the left common femoral vein; **B)** Case 1 - Recanalization of the left femoral vein; **C)** Case 2 - Floating thrombus from the saphenofemoral junction to the left femoral vein; **D)** Case 2 – Complete recanalization of the left saphenofemoral junction; **E)** Case 3 - Floating thrombus at the right saphenofemoral junction; **F)** Case 3 – Complete recanalization of the right saphenofemoral junction. CFV = common femoral vein; GSV = great saphenous vein; L = left; R = right.

**Case 2 -** A 50-year-old male patient presented complaining of edema of the left lower limb with onset 1 week previously after particularly intense physical effort (work-related). He did not exhibit any other associated comorbidities. He reported that he did not smoke or abuse alcohol, had had no previous surgery, immobilization, trauma, obesity, restrictions to walking. He had no family history of PE, DVT, or SVT. On physical examination he was normotensive, with edema of the left lower limb (+++/4) and hyperemia, heat, and pain along the path of the great saphenous vein, from the proximal leg to the proximal thigh of the left lower limb, with no sign of severe chronic venous insufficiency, except for sparse non-communicating varicose veins. Peripheral pulses were palpable and there was muscle hardening in the region of the left thigh and calf. A venous DUS examination of the left lower limb revealed an incompressible great saphenous vein, with no blood flow and with echogenic content (thrombus) in the lumen. The thrombus extended from the mid-leg to the saphenofemoral junction, where it “dived” into the lumen of the common femoral vein and appeared to be “floating” (Figure 1C; Video 1 of Supplementary Material), moving in time with arterial pulsations. Other superficial and deep veins were normal. The diagnostic conclusions of the examination were ascending thrombophlebitis of the great saphenous vein and floating thrombus of the common femoral vein, both in the left lower limb.

**Case 3 -** The patient was a 58-year-old female with varicose veins and long-term chronic venous insufficiency and a venous ulcer of the right lower limb. The ulcer originated from a trauma in the region of the right medial malleolus, with consequent local infection, which was treated using antibiotics (cephalexin). The patient had an allergic reaction to the antibiotics and was obliged to remain at rest, treating the wound with localized dressings. She had intense pain at the ulcer site, worse when standing up. After 15 days of conservative treatment, she sought specialist medical care, at which point she presented with the additional symptoms of hyperemia, pain, and heat along the path of the right great saphenous vein. On clinical examination she was normotensive, distal arterial pulses were present, and she had an active venous ulcer, with granulation tissue, of 4 cm in diameter, on the medial surface of the right leg (CEAP 6). Venous DUS revealed SVT of the right great saphenous vein, extending along its entire path to the saphenofemoral junction, and a floating thrombus was observed at the limit of the saphenofemoral junction, moving with the heartbeat (Figure 1E).

Possible treatment options that were considered for these cases included: anticoagulant treatment^3^^,^ 11; fibrinolytic treatment or thrombolysis^12^; venous ligature or venous plicature^1^; venous thrombectomy^11^; aspirative percutaneous thrombectomy^11^; and inferior vena cava filter (VCF).^11^

## PART II – WHAT WAS DONE

**Case 1 -** The patient was treated with full anticoagulation while in hospital, with a combination of unfractionated heparin and warfarin until the international normalized ratio had been brought within therapeutic levels (INR in the range of 2-3). In view of the PE episode and the floating thrombus, it was decided to implant a removable VCF. A DUS examination of the fourth day after hospital admission showed that the VCF was free from thrombi and well-located, with maintenance of the ultrasonographic status seen previously. He was discharged from hospital after 5 days with a prescription for 5 mg warfarin and instructions to attend routine outpatients follow-up to check anticoagulation levels. At 6 month follow-up, his clinical status was good and a control examination with venous DUS showed total remission from the floating thrombus, with signs of recanalization of the veins that had been involved in the DVT episode (Figure 1B). The VCF was still well-positioned and free from thrombi and was not removed.

**Case 2 -** The patient was given anticoagulation treatment with enoxaparin sodium and warfarin. On the fifth day of treatment he returned for reassessment and exhibited considerable improvement of the edema (+/4) and inflammatory signs. Low molecular weight heparin was withdrawn and the 5 mg warfarin dose was continued, with maintenance of therapeutic INR levels. The patient was prescribed elastic stockings and instructed to return to normal activities. The following week, he returned for another assessment and found to be asymptomatic, with no residual edema, with discretely heightened color along the path of the great saphenous vein in the proximal 1/3 of the leg and the distal thigh. Laboratory tests for thrombophilias were ordered. There were no intercurrent conditions of any type during the initial phase of anticoagulation or up to the time of writing (maintenance phase) and tests for secondary thrombophilias were negative (tests for silent cancer and antiphospholipid antibody syndrome). The venous DUS examination conducted 3 months after the clinical event showed total recanalization of the common femoral vein and the great saphenous vein and total remission of the floating thrombus (Figure 1D).

**Case 3 -** The patient was treated with a combination of 1 mg/kg of enoxaparin every 12h (60 mg) and warfarin while in hospital. Tests for secondary thrombophilias were negative. During outpatients follow-up, therapeutic anticoagulation levels were maintained for 3 months and then anticoagulation was withdrawn. The follow-up venous DUS examination conducted after completion of anticoagulation treatment showed total disappearance of the floating thrombus, recanalization of the great saphenous vein and an absence of signs of DVT (Figure 1F).

## DISCUSSION

In view of the potential autolysis of floating thrombi,^6^ anticoagulant treatment appears to be an attractive noninvasive option, particularly after SVT that has reached the saphenofemoral junction.^13^^,^ 14 Besides the obvious antithrombotic effects, the anticoagulants, especially heparins, also have anti-inflammatory properties that amplify their benefits.^15^ In cases of SVT in isolation, the American College of Chest Physicians (ACCP) 2012 consensus recommended using fondaparinux at prophylactic doses (2.5 mg/day) or low molecular weight heparin for 45 days.^16^ However, when DVT is also present, treatment should be focused on this disease, increasing the duration of anticoagulation. The initial treatment should be provided in a hospital environment. Home treatment should be ruled out because of the high risk of complications related to the possibility of noncompliance with treatment.^17^

The advantages of surgical treatment are: faster treatment of symptoms and shorter hospital stays, which reduces costs.^13^ However, simple ligature of the saphenous trunk does not impede passage of thrombi via perforating and tributary vessels and does not attenuate any hypercoagulable state that may be present.^13^ During surgery, in addition to ligature, it is also recommended that the head of the thrombus that is entering the lumen of the femoral vein should be removed (thrombectomy) by venotomy at the arch of the saphenous vein.^1^ The complications of this type of procedure are hematoma and hemorrhages, the risk of embolizing the thrombus, and recurrence of local thrombosis.^13^ In general, anticoagulant treatment during the postoperative period is recommended.^13^

Thrombolytic treatment effectively dissolves the thrombus and reduces postthrombotic syndrome (RR: 0.66; 95%CI: 0.47-0.94), but it is associated with a higher frequency of hemorrhagic complications.^12^ The ATTRACT study^18^^,^ 19 randomized 692 patients with proximal DVT for fibrinolytic treatment or conventional treatment (anticoagulants and elastic stockings). After 2 years it was observed that fibrinolytic treatment did not prevent postthrombotic syndrome, but did reduce its severity in 25% of cases (18% vs. 24%).^18^

A VCF is indicated if anticoagulants are contraindicated or provoke complications, according to the ACCP consensus.^11^^,^ 16 There are no guidelines for their use in cases of floating thrombi. However, in cases of extensive floating thrombi involving the iliac veins and even the vena cava, use of a VCF could be evaluated, despite the high risk of PE, especially when anticoagulants fail or are contraindicated.

## CONCLUSIONS

There are no specific treatment guidelines for cases of floating venous thrombosis with thrombi that extend into the femoral vein. Anticoagulant treatment appears to be an effective and less invasive option, and surgery can be associated with complications. While there is no consensus, in cases with more extensive floating thrombi, involving iliac veins or even the vena cava, using a VCF could be evaluated, because of the high risk of PE, particularly when anticoagulants are contraindicated.

## Supplementary Material

Supplementary material accompanies this paper.

Video 1 Venous duplex ultrasound of case 2 with floating thrombus at the right saphenofemoral junction. This material is available as part of the online article from http://www.scielo.br/jvb.
